# Clinical Validation of Mobile Cardiotocograph Device for Intrapartum and Antepartum Monitoring Compared to Standard Cardiotocograph: An Inter-Rater Agreement Study 

**Published:** 2019-06

**Authors:** Manoja Kumar Das, Reva Tripathi, Neeraj Kumar Kashyap, Sunita Fotedar, Surender Singh Bisht, Asmita M. Rathore, Aakanksha Raghav

**Affiliations:** 1 The INCLEN Trust International, New Delhi, India; 2 Maulana Azad Medical College, New Delhi, India; 3 Swami Dayanand Hospital, Delhi, India; 4 Hamdard Institute of Medical Sciences and Research, New Delhi, India

**Keywords:** Wireless Cardiotocograph, Fetal Heart Rate, Uterine Contraction, Intrapartum Monitoring, Agreement, Acceptability

## Abstract

**Objective:** Electronic fetal monitoring (EFM) using cardiotocograph (CTG) is commonly used both to assess fetal wellbeing in late antepartum and for intervention during intrapartum period. We validated the performance of indigenously developed mobile cardiotocograph (CTG) device with wireless probes compared to standard CTG device.

**Materials and methods:** We sequentially used mobile and standard CTG devices in 495 pregnant women in labour and 359 pregnant women with gestation > 32 weeks. The CTG interpreted by two independent obstetricians in a blinded manner were compared to estimate the agreement by kappa *(k)* statistic.

**Results:** High level of agreements between mobile and standard CTG devices for both intrapartum (87.9%; *kappa *0.61) and antepartum monitoring (91.2%; kappa 0.60) were observed. Most of the pregnant women (80% in intrapartum and 70% in antepartum groups) and all nurses and obstetricians preferred the mobile CTG device over standard CTG device.

**Conclusion:** The mobile CTG device can reliably be used for both intrapartum and antepartum monitoring instead of the standard CTG devices. The smaller size, portability and ability to transmit the recordings for second opinion make it suitable for use by midwives for appropriate triaging and referral. Wider availability of CTG and interpretation support at the peripheral facilities would assist identifying at-risk pregnancies and foetuses for timely referral and appropriate action to reduce perinatal deaths, stillbirths and birth asphyxi.

## Introduction

Out of the 2.62 million stillbirths globally in 2015, India unacceptably tops the list with contribution of about 592,000 stillbirths (1). Additionally birth asphyxia and intrapartum related events resulted in 631,000 (23.7%) neonatal deaths during 2010-15 globally (2). Intrapartum stillbirth is considered as sensitive marker of quality of intrapartum care. Government of India under the India Newborn Action Plan (INAP), has adopted the target of < 10 per 1000 births by 2030 (3). Foetal heart activity is a key parameter of intrapartum foetal monitoring and indicator of foetal hypoxia. Cardiotocography (CTG) is the commonest method of intrapartum monitoring for foetal heart rate (FHR) and uterinecontractions (UC) used by obstetricians. The available evidences showed no impact of continuous CTG monitoring on cerebral palsy, reduction in neonatal seizures (4). The use of continuous CTG has been associated with rise in instrumental deliveries and caesarean sections (CS) (4).The current guidelines for interpretation of CTG is primarily ‘pattern recognition’, approach, which is at risk for inter- and intra-observer variations (5,6). The wide variation in the clinical practices, standards and methodology adopted and probably the interpretation of CTG in various studies challenge the comparability. Studies have documented high sensitivity (80-100%) of computerised CTG analysis according to FIGO (International Federation of Gynecology and Obstetrics)classification for detection of foetal hypoxia and acidosis (6). Admission test (a short recording of FHR and UC pattern for a period of 20-30 minutes) on entry to labour room has been proposed as a screening test to detect foetuses at risk and need for continuous foetal monitoring (7-9). In addition to the benefit on foetal outcome, majority of the professionals (obstetricians and midwives) prefer foetal surveillance over intermittent auscultation in view of the potential litigations and professional hazards. The intermittent auscultation is also a challenge in several developing country contexts due to poor staffing (10). In view of the dynamic process of labour and unexpected emergency and untoward outcomes, role of foetal monitoring cannot be dispelled till better robust monitoring options are available. There is a global desire to reduce stillbirths and intrapartum asphyxia to reduce the neonatal and infant mortality. Computerised algorithm based interpretation and classification of CTG are being explored to address the intra-and inter-observer variability. The diagnostic tools like CTG and foetal monitoring are available mostly at the referral tertiary care hospitals. Non-availability of such potentially useful tools at peripheral facilities where the availability of professional competence and CTG like devices is limited. We hope that availability of such tools at the peripheral facilities would be useful to identify the at risk foetuses for timely referral and action to reduce the stillbirths and intrapartum hypoxias. Rapid evolution in mobile computing and technological advances have been pushing the boundaries for medical diagnostic technologies and interpretation possibilities (11-13). Under the global effort for Saving Lives at Birth: A Grand Challenge for Development multiple innovations are being explored (14). We report clinical validation of the mobile cardiotocograph device for intrapartum and antepartum foetal wellbeing assessment undertaken under the Grand Challenges program.

## Materials and methods

**Figure 1:**
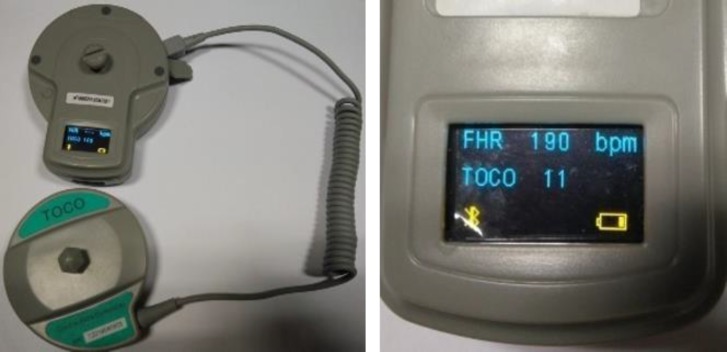
The mobile cardiotocograph device including the probes (FHR and Toco probes)

***Development of the mobile CTG device (mCTG) and assembly: ***The project team in collaboration with CTG device manufacturer developed wireless CTG probe. The wireless probe had a FHR probe (ultrasound Dopplertransducer) with two micro-USB (universal serial bus) ports for connecting the toco-probes (pressure transducer). The probe connects to android mobile program through Bluetooth to send the recorded signals constantly, which is displayed on the mobile screen, also recorded and stored in the mobile device. The FHR probe has inbuilt rechargeable battery, which once charged is able to support continuous recording for about 3-4 hours. Additionally a small screen on the FHR probe provides real-time display of the FHR and uterine contraction tracing along with the battery status. The two probes are attached to the abdomen of the pregnant women similar to the standard CTG probes using elastic bands. The pictures of the probes and screen display on the probe are shown in Figure 1 (1.1-1.2).

1.1 FHR and Toco probes; 1.2 The display screen on FHR probe*

* The screen displays fetal heart rate (FHR) in beats per minute; uterine contraction (Toco) per minute; battery status and bluetooth connectivity status.

The mobile program enables recording, display, storage, review, and printing. As per need the recorded CTG reading can be transmitted to another mobile for review and second opinion. The mobile CTG program was refined based on feedback from users and patients. The device has been registered as mCTG and certified under CE (Conformité Européenne in French which means European Conformity) certification for class I medical devices. 

**Figure 2:**
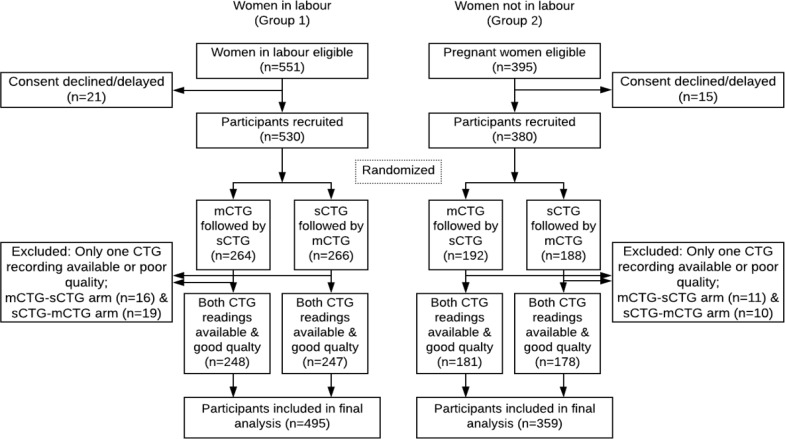
Study participant recruitment and evaluation flow chart

***Validation of the device:*** To assess the reliability of the function of the mCTG device and mobile application, a clinical validation study was undertaken. This observational study adopted crossover study design. The study was conducted during 2016-17, in labour rooms and antenatal clinics of two government teaching tertiary care hospitals in New Delhi. For validation, we compared the readings by the mCTG device with the standard CTG devices (having European CE and United States Food and Drug Administration certification). As simultaneous recording of the standard CTG and mCTG was not possible due to the probe interference, we planned to use the two modes of CTG recording in quick succession. Two groups of participants were recruited after informed written consent: (1) Group 1 (pregnant women in labour) and (2) Group 2 (pregnant women in third trimester attending antenatal clinics, who were not in labour). The inclusion criteria for the group 1 participants were: age 18-45, singleton pregnancy, and gestation age 37-41 weeks. The women with high risk factors or requiring urgent medical or surgical attention were excluded. The inclusion criteria for the group 2 participants were: age 18-45 years, singleton pregnancy, gestation age > 32 weeks, and not in active labour. After obtaining consent the participants in both groups were randomised into two arms: Arm 1 (standard CTG recording followed by mCTG recording) and Arm 2: (mCTG recording followed by standard CTG recording). The random number sequence was generated by an independent biostatistician using alternate blocks of 4 and 6. Allocation concealment was achieved using sequentially numbered triple layered opaque envelopes. For each recruited participants the corresponding unique identity number bearing envelope was opened to assign the arm. The study participant recruitment and evaluation flow chart is shown in figure 2. Each CTG recording was done for at least 20 minutes. The prints of the recordings by both CTG devices were labelled with the unique identity number and archived. For each of the Group 1 participant, information on age, pregnancy, labour, delivery, newborn parameters including birth weight, sex, Apgar score, and resuscitation requirement were collected. For each Group 2 participant, information on age, pregnancy and any high risk factors were collected. All these data were collected using mobile data collection program and uploaded to server on real-time basis. From all participants, feedback about the experience with mCTG and the standard CTG were collected. Data was also collected from the doctor or nurses using the CTG devices about the user experience focusing on the ease of use and challenges.

Multilevel quality assurance and data verification processes (site level verification by supervisor followed by central data management team verification) were adopted. Sixteen independent obstetrician reviewers were invited who underwent a uniform orientation program on reading the CTGs as per FIGO guidelines. Eight pairs of reviewers were made and each pair was assigned the same set of CTGs in a blinded manner; the reviewers were not from the same institution and did not know the other member of their pair. Both CTG readings from the same subject bearing different ID numbers were assigned to same reviewer pair. 

Inter-observer agreement was evaluated by the Cohen’s kappa (k) statistic (15). Landis and Koch’s guidelines were adopted as benchmark scales of ‘k’ coefficients (moderate: 0.41-0.60; substantial: 0.61-0.80 and almost perfect: 0.81-1.0)(16).Statistical analysis was performed using the STATA version 15.0 (Stata Corp LLC, USA). Overall agreement was the proportion of judgments in which two reviewers agreed on categorising the CTG tracing as normal, suspicious and pathological. The Institute Ethics Committees of all three concerned institutes reviewed and approved the study protocol.

## Results

A total of 530 participants in Group 1 and 380 participants in Group 2 were recruited. One of the CTG recording was not available in 35 participants Group 1 and 21 participants in Group 2. Thus analysis included 495 participants in Group 1 and 359 participants in Group 2, who had both mCTG and standard CTG recordings available. The flowchart for recruitment and evaluation is shown in figure 2. The demographics, pregnancy and foetal outcomes for the participants are summarised in table 1. 

***Agreement between CTG readings in Group 1:*** In 87.9% of the CTG recordings from women in labour, the reviewers agreed on the category classification of the CTGs with kappa (k) coefficient of 0.61 (95% CI 0.52, 0.70). The overall and category specific agreements between the CTG readings for Group 1 by standard CTG and mCTG devices are reflected in table 2.

***Agreement between CTG readings in Group 2:*** The overall Kappa *(k)* coefficient for the CTG recordings from women not in labour was 0.60 (95% CI0.47- 0.74). Both reviewers agreed on the category classification in 91.9% cases. The overall and category specific agreements between the CTG readings by standard CTG and mCTG devices for Group 2 are reflected in table 3.

***Participant feedback:*** On asking about the experience of the CTG devices and preference if both were available, 394 (80%) of the Group 1 women expressed preference for the mCTG device, followed by 79 (16%) open to any of the devices and 22 (4%) who preferred standard CTG device.

**Table 1:** Demography characteristics of the participants and the pregnancy outcomes

	**Group 1 (Intrapartum)** **(n = 495)**	**Group 2 (Antenatal)** **(n = 359)**
Age (completed years)	25 (95% CI: 21, 32)	24 (95 CI: 20, 32)
Gestational Age (weeks)	39.2 (95% CI: 37.2, 41.3)	36.4 (95% CI: 34.1, 38.5)
Gravida		
1	260 (52.5%)	163 (45.4%)
2	153 (31.1%)	122 (34%)
≥ 3	82 (16.4%)	74 (20.6%)
Para		
0	283 (57.2%)	181 (50.5%)
1	152 (30.7%)	134 (37.4%)
≥ 2	60 (12.1%)	44 (12.1%)
Mode of delivery		
Normal vaginal	411 (83.1%)	
Assisted vaginal	13 (2.6%)	
Caesarean section	71 (14.3%)	
Fetal outcome		
Live birth	480 (97%)	
Female	236 (47.7%)	
Birth weight (grams)	2800 (95% CI: 2105, 3620)	

**Table 2:** Agreement between standard CTG and mobile CTG recordings for women in labour (Group 1)

		**Standard CTG (n)**				**Total observed ** **agreement (%)**	**Total expected ** **agreement (%)**	**Kappa** ***k*** ** (95% CI)**
		**Normal**	**Suspi-** **cious**	**Patho-** **logical**	**Total**			
mCTG (n)	Normal	376	23	10	409	88.7	70.0	0.62 (0.53-0.71)
	Suspicious	19	39	3	61	90.7	78.1	0.57 (0.45-0.69)
	Pathological	4	1	20	25	96.4	89.0	0.67 (0.521-0.82)
	Total	399	63	33	495	87.9	68.5	0.61 (0.52-0.70)

Among the Group 2 participants, while 250 (70%) preferred mCTG device, 55 (15%) preferred standard CTG device and 54 (15%) were open for any one. The reasons for preference of mCTG mentioned were small size and no wires, which allowed mobility.

***User feedback: ***We obtained responses from 32 nurses and 8 obstetricians at these hospitals who used the devices. All of the users could use the devices without any problem in handling. The connectivity with the probe of the mobile application was smooth and could catch the signals up to a distance of 7-8 meters. The experience about navigation through the mobile application was smooth. Battery backup of 4-5 hours for the probes following one full charge was observed. The data backup and retrieval from the mobile and server was easy, which was considered as an advantage in addition to the print facility.

## Discussion

According to the available literature reviewed by us, the current study is the first effort to explore agreement between a mobile CTG device with standard CTG device for both intrapartum and antenatal periods. CTG is being used by obstetricians globally for monitoring and predicting the potential foetal outcomes and decision making tool for mode of delivery. Admission CTG test has been also used as triage tool for pregnant women in labour though it has not attained widespread acceptance. The standard CTG devices are comprised of wired probes, power dependent, relatively bulkier and also costly limiting the availability at the lower level public health facilities. Additionally these devices also limit the mobility of the patients during the period of recording though new wireless devices are available but their higher cost is a further deterrent. The current study observed high levels of overall agreement (87.9%-91.9%) between the standard and mobile CTG device readings with substantial* k* statistic of > 0.6. Despite a high proportion of agreement, lower kappa values may be observed, when the marginal values are imbalanced. On the contrary, higher kappa value may be observed for asymmetrical imbalanced marginal totals. Kappa is affected by prevalence and may not be reliable for rare observations. Thus very low values may not necessarily reflect low overall agreement. Decision on performance of a tool should also consider the observed *versus* expected agreement, consistency across contexts, and suitability of the criteria for specific settings besides kappa statistics (17, 18).

These observations indicate good performance of the mobile CTG device compared to the standard CTG devices and very good acceptance by both patients and users. The advantages of mobile CTG device and probe are the small size, absence of wires, facilities for data storage and retrieval and subsequent possibility of transfer for second opinion. The program can be loaded on any android mobile or tablet for accessing the readings. These features make the mobile CTG suitable for use in public and private health system and integration with other mobile program platforms for health care service delivery.

**Table 3:** Agreement between standard CTG and mobile CTG recordings for women not in labour (Group 2)

		**Standard CTG (n)**				**Total observed ** **agreement (%)**	**Total expected ** **agreement (%)**	**Kappa** ***k*** ** (95% CI)**
		**Normal**	**Suspi- ** **cious**	**Pat,./ho** **-logical**	**Total**			
mCTG (n)	Normal	306	9	4	319	93.0	80.0	0.65 (0.52-0.78)
	Suspicious	8	17	2	27	94.2	85.9	0.58 (0.41-0.75)
	Pathological	4	2	7	13	96.7	93.0	0.52 (0.25-0.78)
	Total	318	28	13	359	91.9	79.4	0.60 (0.47-0.74)

The proportion of normal, suspicious and pathological readings at admission observed were 82.6-85.8%, 12.3-12.7% and 5-6.6% respectively according to different CTG device recordings. These proportions were comparable to the admission test findings reported in recent literature from India (   7 -11). Wireless foetal monitors (like Moyo) are being explored for documenting and monitoring FHR in developing countries (21). But no information on uterine contraction status limit the acceptance by the doctors and obstetricians.

The crossover study design using both devices in the same participant in intrapartum and antepartum setting with adequate sample sizes is the strength of the study. Two CTG devices could not be used for simultaneous recording in same subject due to probe field interferences and we had to adopt a sequential recordings using two CTGs. The variation in FHR and UC patterns during the progressing labour captured by the two different CTG devices might have changed the classification of CTG and interpretation by different obstetricians. In view of this, the real agreement between the devices may be higher than observed in the current study. The study did not document the impact of CTG on intrapartum decision making as these readings were not utilised for the same and independent decisions were taken by the treating obstetricians.

## Conclusion

In conclusion, performance of the new mobile CTG device was as good as the standard CTG device with several improved features and advantages. The small size, portability, connectivity, ease of use, good battery backup may offer advantage for use at all types of health facilities. The ability to send the CTG tracing for confirmation or second opinion is a useful tool for non-obstetrician physicians for appropriate decision making. Better acceptability of mCTG by patients compared to standard device is encouraging. Availability of the mCTG or similar device for pregnant women at peripheral facilities may allow assessing the impact on perinatal deaths, stillbirths, birth asphyxias and early neonatal deaths.

***Compliance with ethical standards:*** This study protocol was reviewed and approved by the institute ethics committees of the participating institutes; The INCLEN Ethics Committee, New Delhi (protocol ref no IIEC 024), Maulana Azad Medical College, New Delhi (F.1/IEC/MAMC/54/3/2016/No117) and Swami Dayan and Hospital, New Delhi (SDNH/HIEC/3). 

Informed written consent was obtained from all the individual study participants before recruitment into the study. All procedures performed in the study were in accordance with the ethical standards of the institutional research committees, Ethical Guidelines for Biomedical Research on Human Participants by Indian Council of Medical Research and with the 1964 Helsinki declaration and its later amendments. This article does not contain any studies with animals performed by any of the authors.

***Funding support:*** The project was supported by Grand Challenges Canada, Toronto, Canada (grant number 0700-03) under the Saving Lives at Birth Grand Challenge (Round 4). The funder provided finance support only and had no role in study design, implementation, interpretation, report writing, decision to publish the article.
